# Supplementary material for: Targeting androgen receptor as a novel radiosensitizing therapy to improve long-term survival and anti-tumor immunity in glioblastoma via TGF-β/Smad3 Axis reprogramming
Source: Cell Death Discov. 2026 Jul 25;12:421. doi: 10.1038/s41420-026-03259-9 (PMC13683371; doi:10.1038/s41420-026-03259-9)
Supplement: Supplementary file 1 [file 41420_2026_3259_MOESM1_ESM.docx]

**Supplementary Information**

**Targeting Androgen Receptor as a Novel Radiosensitizing Therapy to Improve Long-Term Survival and Anti-tumor Immunity in Glioblastoma *via* TGF-β/Smad3 Axis Reprogramming**

^1, 2^Jyoti B. Kaushal, Ph.D.; ^2^Nan Zhao, M.B.B.S.; ^2^Rubayat Khan, Ph.D.; ^3^Kan Liu, Ph.D.; ^3^Chi Zhang, Ph.D.; ^2^Fei Wang, M.B.B.S.; ^4^Jie Chen, M.D., Ph.D.; ^5^Bingjie Guan, B.S.; ^2^Shuo Wang, Ph.D.; ^6^Tom K. Hei, Ph.D.; ^7^Tony J. C. Wang, M.D.; ^8^Yuguo Lei, Ph.D.; ^9^Michele R. Aizenberg, M.D.; ^1^Chi Lin, M.D., Ph.D.; *^1,2^Chi Zhang, M.D., Ph.D.

*^1^Department of Radiation Oncology, Mayo Clinic Arizona, Phoenix, AZ, USA*

### ***^2^****Department of Radiation Oncology, Fred & Pamela Buffett Cancer Center, University of Nebraska Medical Center, Omaha, NE, USA*

*^3^School of Biological Sciences, University of Nebraska - Lincoln, Lincoln, NE, USA*

*^4^Department of Pathology, University of Nebraska Medical Center, Omaha, NE, USA*

*^5^Department of Radiation Oncology, United Hospital of Fujian, Fuzhou, Fujian, P.R. China*

*^6^Center for Radiological Research, College of Physicians and Surgeons; Columbia University, New York, New York, NY, USA*

*^7^Department of Radiation Oncology, Columbia University Irving Medical Center, New York, New York; Herbert Irving Comprehensive Cancer Center, New York, NY, USA*

*^8^Department of Biomedical Engineering, Huck Life Science Institute, Pennsylvania State University, University Park, PA, USA*

### ***^9^****Department of Neurosurgery, Fred & Pamela Buffett Cancer Center, University of Nebraska Medical Center, Omaha, NE, USA*

***Corresponding author**

Chi Zhang, M.D., Ph.D.

Department of Radiation Oncology,

Mayo Clinic Arizona

Phoenix, AZ 85054

Email: [zhang.chi3@mayo.edu](mailto:zhang.chi3@mayo.edu); Phone: 480-342-3971

**Running title:** AR Blockade Plus RT Prolongs GBM Survival, Boost Immunity

**Supplementary Figures**

**Fig. S1:** **Effect of ARAs on human GBM cells, normal cortical neurons, astrocytes, and primary HGG cells, and their combined treatment with radiation on colony formation and spheroid growth. (A)** Cell viability was assessed using the CTB assay in U87MG cells treated with various concentrations of ARAs (ENZA or BIC) and in combination with radiation doses (8 Gy or 16 Gy). Data are expressed as mean ± SEM (n = 5). Statistical significance was defined as P ≤ 0.05. For group comparisons, *P < 0.05, **<0.01, ***<0.001 for treated group vs. untreated control cells. **(B)** Representative images show morphological alterations and reduced cell growth across the different treatment groups as presented, in human primary HGG cells**. (C)** Cell viability was determined by CTB assay in normal human cortical neuronal (HCN2) and astrocyte (ASTRO) cells treated with different concentrations of AR antagonists (ENZA or BIC). Results are shown as mean ± SEM (n = 5). **(D, E)** Visualization of colony formation capabilities in human GBM cells (U87MG) and primary cultured HGG cells treated with ARAs in combination with varying radiation doses (2 Gy, 4 Gy, and 8 Gy). **(F)** Representative images showing the day-dependent growth of human primary HGG cell spheroids treated with AR antagonists (ENZA, 40 µM; BIC, 80 µM) alone, 16 Gy radiation alone, their combination, or untreated control.

**Fig. S2.** **Effect of AR antagonists (ARAs) on apoptosis and cell cycle alteration in various human GBM cell lines: (A)** The representative figures of cell apoptosis analysis by flow cytometry in U87MG, Ln229, and U138MG cell lines after the treatment of DMSO or ENZA for 2, 3, or 4 days (upper panel). Percentages of cells in early apoptotic phase, late apoptotic phase and dead cells of these cell lines are presented. * p<0.05; **p<0.01; ***p<0.001; ****p<0.0001 (lower panel). **(B)** Representative results of cell cycle analysis by flow cytometry on U87MG, U138MG, and Ln229 cells treated with ENZA (80 μM) for 48 h (upper panel). Quantitative analysis of cell cycle distributions of these micrographs was shown (***p<0.001; ****p<0.0001 group) (lower panel).

 **Fig. S3.** **Rechallenging of the long-term survivors of mice after previous ARA or ARA+RT treatment with the second time of MGPP3 cell implant to contralateral side of brain.** (A) Representative IVIS images of the tumor growth in mouse brain after rechallenging. Wk 1, 3 and 4: IVIS imaging taken 1, 3 and 4 weeks after initiation of MGPP3 cell implantation. Newly injected mice: mice with the first time implantation of MGPP3 cells as positive control; Bicalutamide: mice survived for a long term after the first time tumor implantation with bicalutamide treatment were re-challenged with MGPP3 cell implantation the second time 260 days after the first time implantation; bicalutamide+RT: mice survived for a long term after the first time tumor implantation with bicalutamide+RT treatment were re-challenged with MGPP3 cell implantation the second time 260 days after the first time implantation. (B) Overall survival curves of the mice that survived with complete radiographic tumor response after bicalutamide or bicalutamide plus RT treatment after the first time of tumor implantation and were rechallenged with the second time MGPP3 cell implantation in the brain. After the rechallenge, the mice were followed for another 50 days with no brain tumor developed per IVIS imaging and all survived. No long-term survivors were from vehicle (control) or RT only treatment group after the first-time tumor implantation thus no re-challenge was done. Dashed line arrow: the time point of the second time tumor implantation.

**Fig. S4.** mRNA expression level of AR positively correlates with the levels of many genes involved in TGF-β pathway in TCGA database. *: p<0.05; **: p<0.01; ***: p<0.001; ****: p<0.0001.

**Fig. S5.** Comparison of mRNA expression levels in TGF-β pathway genes between GBM and normal brain tissues and the correlations between mRNA and protein expression levels in the representative genes in GBM. (A-I) mRNA expression levels of AR (A), smad2 (B), smad3 (C), smad4 (D), smad6 (E), smad7 (F), TGF-β1 (G), TGF-β2 (H), and TGF-β3 (I) in GBM and normal brain. (J) correlations between mRNA and protein expression levels for AR, smad3, and smad4 in GBM in TCGA database. *P < 0.05.

**Fig. S6. Smads/Stats gene expression, Smad3 subcellular localization, and p-Smad3C/TGF-β2 levels following ENZA treatment, radiation, or their combination. (A)** mRNA expression level changes of smads and stats before and after the treatment of ENZA for 0 (control), 8, 24, and 48 hours. *P < 0.05, **P < 0.01. **(B)** Representative confocal laser scanning microscopy images displaying the subcellular expression patterns of Smad3 in U87MG cells treated with 20 µM or 80 µM ENZA for 24 hours (upper panel). The quantitative fluorescence intensities of the confocal images are shown for the designated groups (n = 3) (lower panel). **(C)** Representative confocal images showing the expression and subcellular localization of TGF-β2 and pSmad3C(S423/425) in U87MG cells treated with ENZA (20 µM) alone, radiation alone (2Gy, 4Gy, 8 Gy, and 16 Gy), or in combination with ENZA.

**Fig. S7. Flow cytometry of immune cells in peripheral blood from the rechallenged mice with or without pretreated bicalutamide ± RT.** Percentage of CD4+ and CD8+ T cells, percentage of CD4+ and CD8+ T naive cells, and percentage of CD4+ and CD8+ T effector memory cells are shown (upper panel) and percentage of CD4+ and CD8+ regulatory T cells (Treg), percentage of CD4+ and CD8+ T central memory cells and percentage of CD4+ and CD8+ T effector cells are shown (lower panel). Analysis was performed in the peripheral blood of the mice 4 weeks after tumor rechallenging. *P < 0.05.

**Fig. S8**. **Flow cytometry analysis of T lymphocyte subtypes after tumor rechallenge in mice with prior bicalutamide and/or radiation treatment.** Example of flow cytometry for comparison of subtypes of T lymphocytes in spleen tissue from mice that were rechallenged with tumor cell implantation after previously being treated with bicalutamide only, bicalutamide plus brain RT (BIC+RT), or in those without previous treatment (control). Left column showed the methods of subtyping of T lymphocytes from mononuclear cells from the spleen. Fluorescence Minus One (FMO) for CD44 or CD62L was performed as shown on the top right panels for control purposes.

**Supplementary Material and Methods**

**Syngeneic orthotopic GBM mouse model**

5 x 10^4^ MGPP3 murine GBM cells were implanted into the right brain hemisphere of 16 to 17-week-old male mice weighing 20 to 30 g. The growth of the tumor was monitored using PerkinElmer *In vivo* Imaging System (IVIS) each week as previously reported ^1^. The mice were regrouped into vehicle (negative control) (10% DMSO, 30% PEG400, 60% corn oil), enzalutamide (ENZA) treatment only, bicalutamide (BIC) treatment only, RT only, ENZA plus RT, and BIC plus RT treatment groups with equivalent tumor sizes based on mean values of bioluminescence signals from brain tumor between groups at week 5 after the implantation. ENZA (20 mg/kg), BIC (60 mg/kg) or vehicle only (100 µl/injection) were injected intraperitoneally (IP) into the mice three times per week following the published protocols ^2^. Two fractions of RT (10 Gy/fraction) were delivered to the mouse brain one fraction per week with dose regimen selected to achieve ~50% of survival. The first fraction of RT was given one week after the initiation of ENZA/BIC treatment.

ENZA or BIC was IP injected continuously to the mice until Week 15 after the implantation (week 10 after starting drug treatment) or death. The mice presented with signs of near death such as seizures were euthanized with neck dislocation. After death was confirmed, mice were perfused with 10% formalin in PBS and brain tissues were dissected for immunohistochemistry (IHC) studies. For the long-term survivors, tumor was rechallenging at day 260 after the initial tumor inoculation. All studies were carried out in compliance with the local ethical guidelines for animal experiments. The protocol was approved by the Institutional Animal Care and Use Committee (IACUC) of University of Nebraska Medical Center (protocol #: 16-134-01). All the mice with tumor received palliative care for pain control after surgery, during the follow-up and prior to euthanization per institutional guidelines.

**Cell cycle analysis**

PI/RNase Staining Buffer (BD Biosciences, San Jose, CA, USA) was used to analyze the cell cycle distribution as previously described^3^. U87MG, Ln229 and U138MG cell lines were treated with DMSO or ENZA (80 μM) for 48 hours before harvesting with trypsin. Cells were washed with PBS and fixed in cold 70% ethanol for 30 minutes at 4 °C. After washing twice with PBS, cells were resuspended in 200 μl of 50 μg/ml propidium iodide (PI) and analyzed with FACS LSRII G Flow Cytometer (Beckon-Dickinson, Franklin Lakes, NJ, USA). The cell cycle distribution was analyzed with ModFit LT software (Verity Software House, Topsham, ME).

**Cell apoptosis assay**

Apoptosis in GBM cell lines following ENZA treatment was assessed using Annexin V conjugated with APC (Annexin V-APC) and propidium iodide (PI) (BD Biosciences, San Jose, CA, USA) in accordance with a previously published method ^3^. U87MG, U138MG and Ln229 cells were seeded into 6-well plates and treated with DMSO or ENZA for different days. All the cells were collected in a tube and stained with Annexin V-PE for 15 minutes at room temperature in the dark. After adding PI, all the samples were analyzed with FACS LSRII G Flow Cytometer (Beckon-Dickinson, Franklin Lakes, NJ, USA). Data were analyzed by FACSDiva software (Beckon-Dickinson, Franklin Lakes, NJ, USA).

**Magnetic Resonance Imaging (MRI)**

The mice treated with vehicle, BIC, RT, and BIC plus RT had MRI imaging of the brain 5 weeks after completing all treatment. Mice were anesthetized by inhalation of 1.5% isoflurane in a 70% nitrous oxide/30% oxygen mixture prior to MRI acquisition and followed the previously published method ^4^. 3D high resolution T_1_-weighted (T_1_-wt) MRI scan was acquired from each animal using the Fast Low Angle Shot (FLASH) sequence followed by a 3D high resolution T_2_-weighted (T_2_-wt) Rapid Acquisition with Relaxation Enhancement (RARE) sequence in a Bruker (Karlshure, Germany) 7 T MRI system operating Paravision 4.0. The acquisition parameters used were TR = 800 ms, effective TE = 30 ms, RARE factor = 16, averages = 1 for a total acquisition time of 8.5 min. In both sequences, the field of view (FOV) was 20×20×20 mm with a matrix of 256×128×128 (voxel size = 78×150×150 μm). The reconstruction matrix size was 256 isotropic (voxel size = 78 μm isotropic) for both scans.

**Flow cytometry of tumor-infiltrating immune cells from mouse tissues**

For flow cytometry analysis of tumor-infiltrating immune cell populations in the animal study, 4 weeks after implantation, mice were sacrificed with cervical spine dislocation. Mouse brains were removed and gently minced using a clean scalpel. The brain tissue was dissociated with 3ml Accutase solution for 40 minutes at 37 °C with occasional resuspension. Dissociation was stopped by adding 5ml of prewarmed PBS/10%FBS. Cells were then filtered through a 40-μm cell strainer (BD Falcon), centrifuged at 400 g, and resuspended in 4 ml of 30% Stock Isotonic Percoll. The cells were overlaid onto a Percoll gradient (30%/37%/70%) and centrifuged at 800g for 20 minutes. Lymphocytes were collected from the 37%/70% interphase, washed twice in PBS, then counted and stained with antibodies in conjunction with Mouse BD Fc Block and viability dye. Spleens were harvested from each mouse, and splenocytes were collected by homogenization of the whole spleen followed by incubation in red blood cell lysis buffer for 5 minutes. Mice peripheral blood was harvested and lysed by red blood cell lysis buffer for 5 minutes at room temperature. Cells were then washed in PBS and counted. For the flow cytometry experiment, 3x10^6^ cells per tube were labeled with antibodies, as displayed in Table S3. All acquisition was performed by a BD LSR II Green flow cytometer and followed the published method ^5,6^. Data analysis was performed via FlowJo software. Naïve T cells are defined as CD3+/CD44^low^/CD28^high^/CD62L^high^, central memory T cells (Tcm) are defined as CD3+/CD44^high^/CD28^high^/CD62L^high^, effector memory cells (Tem) are defined as CD3+/CD44^high^/CD28^high^/CD62L^low^, effector T cells/terminally differentiated effector memory T cells are defined as CD3+/CD44^low^/CD28^low^/CD62L^low^.

**Quantification of bicalutamide concentrations in tissues with Ultra-Performance Liquid Chromatography (UPLC)**

In a 1.5 mL test tube, 100 μL of collected plasma specimen or 50 mg of brain tissue (homogenized in 500 µl HPLC grade water) were added to 1ml acetonitrile, containing 20µl Tolbutamide (15µg/ml). After vortexing for one minute, all samples were centrifuged at 18,500 RCF for ten minutes at 4°C. The supernatants were collected and transferred to a new tube for evaporation to dryness. The dried residues were reconstituted with 100µl of mobile phase solution of acetonitrile: water (50:50, v/v) and drug contents were analyzed by UPLC.

The UPLC data acquisition was carried out on a Waters UPLC BEH C18 column (2.1 mm × 50 mm, 1.7 μm) (Milford, MA) at a temperature of 30°C. The mixture of acetonitrile and water (50:50, v/v) at isocratic elution was used as mobile phase at flow rate of 0.6ml/minute. Calibration curve of Bicalutamide was prepared in the range from 1.56µg/ml to 100µg/ml, using Tolbutamide as an internal standard. Spiking concentration of Tolbutamide was 15µg/ml for this study. The injection volume used for standards and samples was 1µl.

**Real-time growth kinetics**

Cells were seeded in 96-well plates and treated with the indicated drugs, as shown in the representative graph, for growth analysis and for analysis followed the published method ^7^. Phase objects were quantified in real time using the IncuCyte integrated analysis software (Sartorius) to monitor cell growth. Phase-contrast images acquired from the same wells were used to normalize and quantify cell proliferation/growth using the IncuCyte Cell-by-Cell Analysis software module (Sartorius). The data were exported to Excel, and growth at specific time points was measured and quantified.

**References**

1.	Zhao N, Wang F, Ahmed S, Liu K, Zhang C, Cathcart SJ, et al. Androgen Receptor, Although Not a Specific Marker For, Is a Novel Target to Suppress Glioma Stem Cells as a Therapeutic Strategy for Glioblastoma. Front Oncol 2021, 11: 616625–616625.

2.	Sonabend AM, Bansal M, Guarnieri P, Lei L, Amendolara B, Soderquist C, et al. The transcriptional regulatory network of proneural glioma determines the genetic alterations selected during tumor progression. Cancer Res 2014, 74(5): 1440–1451.

3. Kaushal JB, Bhatia R, Kanchan RK, Raut P, Mallapragada S, Ly QP, et al. Repurposing Niclosamide for Targeting Pancreatic Cancer by Inhibiting Hh/Gli Non-Canonical Axis of Gsk3beta. Cancers (Basel) 2021, 13(13).

4.	Uberti MG, Boska MD, Liu Y. A semi-automatic image segmentation method for extraction of brain volume from in vivo mouse head magnetic resonance imaging using Constraint Level Sets. J Neurosci Methods 2009, 179(2): 338–344.

5.	Benichou G, Gonzalez B, Marino J, Ayasoufi K, Valujskikh A. Role of Memory T Cells in Allograft Rejection and Tolerance. Front Immunol 2017, 8: 170.

6.	Zhang X, Chang Li X, Xiao X, Sun R, Tian Z, Wei H. CD4(+)CD62L(+) central memory T cells can be converted to Foxp3(+) T cells. PLoS One 2013, 8(10): e77322.

7	Kaushal JB, Raut P, Halder S, Alsafwani ZW, Parte S, Sharma G, et al. Oncogenic potential of truncated-Gli3 via the Gsk3beta/Gli3/AR-V7 axis in castration-resistant prostate cancer. Oncogene 2025, 44(15): 1007–1023.

**Supplementary Tables**

**Table S1:** The cell lines used in this study are as follows.

**Table S2:** List of antibodies and other relevant details utilized in this study.

| **REAGENT or RESOURCE** | **SOURCE** | **IDENTIFIER** |
| --- | --- | --- |
| **Primary Antibodies -WB/IF** | | |
| Anti- p-Stat3 (Tyr705) antibody | Cell Signaling Technology Inc. | Cat# 9131 |
| Anti- pStat3 (Ser727) antibody | Cell Signaling Technology Inc. | Cat# 44-384G, 1:100 |
| Anti- Stat3 antibody | Abcam | Cat# ab109085 |
| Anti- LIF antibody | Abcam | Cat# ab138002 |
| Anti- LIFR antibody | Abcam | Cat# ab202847 |
| Anti- PIAS3 (D5F9) antibody | Cell Signaling Technology Inc. | Cat# 9042T |
| Anti- pSmad3C(S423/425) antibody | Abcam | Cat# ab52903 |
| Anti- pSmad3C(S423/425) antibody | Thermo Fisher Scientific Inc | Cat#44-246G |
| Anti- anti-pSmad3 (S213) antibody | Thermo Fisher Scientific Inc. | Cat# PA5-12694 |
| Anti- pSmad3 (S245) antibody | Thermo Fisher Scientific Inc. | Cat# 44-246G |
| Anti- Smad3 antibody | Abcam | Cat# ab40854 |
| Anti- Smad2 (S467) antibody | Abcam | Cat# ab53100 |
| Anti- Smad2 (S245) antibody | Cell Signaling Technology Inc. | Cat# 3104 |
| Anti- Smad2 antibody | Abcam | Cat# ab40855 |
| Anti- β-actin antibody | MilliporeSigma | Cat# AC-74 |
| Anti- β-actin antibody | MilliporeSigma | Cat# A5316 |
| **Secondary Antibodies -WB** |  |  |
| Goat Anti-Rabbit LgG (H+L) | Thermo Fisher Scientific Inc | Cat# 31460 |
| Beta Actin secondry-HRP conjugated | Thermo Fisher Scientific Inc | Cat# 31470/VB292786 |
| IRDye 800CW Goat Anti-Mouse LgG (H+L) | Li-Cor Biotechnology | Cat# 926-32210 |
| **Secondary Antibodies -IF** |  |  |
| Goat anti-Mouse IgG (H+L) Secondary Antibody, Alexa Fluor 568 | Invitrogen | Cat# A-11004 |
| Goat anti-Mouse IgG (H+L) Secondary Antibody, Alexa Fluor 488 | Invitrogen | Cat# A-11001 |
| **Primary Antibodies -IHC** |  |  |
| Anti- CD8a antibody | Thermo Fisher Scientific Inc | Cat# 14-0808-82 |
| Anti-CD4 antibody | Abcam | Cat# ab183685 |
| Anti-CD68 antibody | Abcam | Cat# ab125212 |
| Anti-FoxP3 antibody | Abcam | Cat# ab215206 |
| **Inhibitors and others** |  |  |
| ITD-1 | MedChemExpress | Cat# HY-12704 |
| Human Recombinant TGFβ2 protein | Cell Signaling | Cat#27615S |
| Human TGFB2 ELISA Kit | Thermo Fisher Scientific Inc | Cat#EHTGFB2 |
| **Software and algorithms** | | |
| ImageJ | ImageJ | <https://imagej.net/software/imagej/> |
| GraphPad Prism 10 | GraphPad | <https://www.graphpad.com/> |
| Sartorius/Incucyte 2022B rev2 | Incucyte | Imaging and analysis software |

**Table S3**. Antibodies used for flow cytometry

**Table S4.** Measured bicalutamide drug distributions in different tissues in male mice with orthotopically implanted GBM after 7 weeks of drug treatment. UPLC analyses of bicalutamide concentrations were conducted in brain hemisphere without tumor (normal brain), the hemisphere where GBM was implanted (tumor brain), liver, pancreas and plasma. *:µg/ml for plasma only.

*
