# Supplementary material for: Targeting androgen receptor as a novel radiosensitizing therapy to improve long-term survival and anti-tumor immunity in glioblastoma via TGF-β/Smad3 Axis reprogramming
Source: Cell Death Discov. 2026 Jul 25;12:421. doi: 10.1038/s41420-026-03259-9 (PMC13683371; doi:10.1038/s41420-026-03259-9)
Supplement: Supplementary file 2 [file 41420_2026_3259_MOESM2_ESM.docx]

**­SUPPLEMENTARY FILE (FULL BLOTS:** **HIGHLIGHTED AREA)**

**Targeting Androgen Receptor as a Novel Radiosensitizing Therapy to Improve Long-Term Survival and Anti-tumor Immunity in Glioblastoma *via* TGF-β/Smad3 Axis Reprogramming**

^1, 2^Jyoti B. Kaushal, Ph.D.; ^2^Nan Zhao, M.B.B.S.; ^2^Rubayat Khan, Ph.D.; ^3^Kan Liu, Ph.D.; ^3^Chi Zhang, Ph.D.; ^2^Fei Wang, M.B.B.S.; ^4^Jie Chen, M.D., Ph.D.; ^5^Bingjie Guan, B.S.; ^2^Shuo Wang, Ph.D.; ^6^Tom K. Hei, Ph.D.; ^7^Tony J. C. Wang, M.D.; ^8^Yuguo Lei, Ph.D.; ^9^Michele R. Aizenberg, M.D.; ^1^Chi Lin, M.D., Ph.D.; *^1,2^Chi Zhang, M.D., Ph.D.

*^1^Department of Radiation Oncology, Mayo Clinic Arizona, Phoenix, AZ, USA*

### ***^2^****Department of Radiation Oncology, Fred & Pamela Buffett Cancer Center, University of Nebraska Medical Center, Omaha, NE, USA*

*^3^School of Biological Sciences, University of Nebraska - Lincoln, Lincoln, NE, USA*

*^4^Department of Pathology, University of Nebraska Medical Center, Omaha, NE, USA*

*^5^Department of Radiation Oncology, United Hospital of Fujian, Fuzhou, Fujian, P.R. China*

*^6^Center for Radiological Research, College of Physicians and Surgeons; Columbia University, New York, New York, NY, USA*

*^7^Department of Radiation Oncology, Columbia University Irving Medical Center, New York, New York; Herbert Irving Comprehensive Cancer Center, New York, NY, USA*

*^8^Department of Biomedical Engineering, Huck Life Science Institute, Pennsylvania State University, University Park, PA, USA*

### ***^9^****Department of Neurosurgery, Fred & Pamela Buffett Cancer Center, University of Nebraska Medical Center, Omaha, NE, USA*

***Corresponding author**

Chi Zhang, M.D., Ph.D.

Department of Radiation Oncology,

Mayo Clinic Arizona

Phoenix, AZ 85054

Email: [zhang.chi3@mayo.edu](mailto:zhang.chi3@mayo.edu); Phone: 480-342-3971

**Running title:** AR Blockade Plus RT Prolongs GBM Survival, Boost Immunity

**Figure 4F**

**Figure 4 G**

**Figure 5 D**

**Figure7 (B)**

**Figure7 (F)**

**Figure7 (G)**
